# Supplementary material for: Quantifying Retail Agglomeration using Diverse Spatial Data
Source: Sci Rep. 2017 Jul 14;7:5451. doi: 10.1038/s41598-017-05304-1 (PMC5511147; doi:10.1038/s41598-017-05304-1)
Supplement: Supplementary file 1 — supplementary_information [file 41598_2017_5304_MOESM1_ESM.pdf]

Quantifying Retail Agglomeration Using Diverse Spatial Data
Supplementary Information

Duccio Piovani , Vassilis Zachariadis, Michael Batty

1 Datasets

In this section, we will trawl the datasets we have used to calibrate and validate the model. These are a combination of formal proprietary datasets of travel behavior and economic activity, and passively collected data sources of digital social media footprints.

London Travel Demand Survey. LTDS is a continuous household survey of the London area, covering the London boroughs as well as the area outside Greater London but within the M25 motorway. Results in the most recent report relate to residents of the Greater London area, comprising the 32 London boroughs and the City of London. The first year of results covered the financial year 2005/06, meaning that there are now eight years of data available. The survey is a successor to the household survey component of the London Area Transport Survey (LATS) which was last carried out in 2001. The LTDS annual sample size is around 8,000 households in a typical year, a sum of 65,000 households for the 2005-2013 period. LTDS captures information on households, people, trips and vehicles. All members of the household are surveyed, with complete trip detail for a single day recorded for all household members aged 5 and over. Three questionnaires are used for the household questionnaire, individual questionnaires for all household members, and trip sheets for travel diaries. The latter capture data on all trips made on a designated travel day, the same day for all members of the household. Details captured include trip purposes, modes used, trip start and end times, and the locations of trip origins and destinations. We have used this dataset only to calibrate the parameter in the cost function.

Valuation Office Business Rates. Since, the recent online publication of the Valuation Office Agency Business Rates for 2005 and 2010, the business rates of all business premises in England and Wales have become available to the public and offer a unique in-depth extent and geographic precision dataset. VOA compiles and maintains lists of rateable values of the 1.7 million non-domestic properties in England, and the 100,000 in Wales, to support the collection of around a £25 billion in business rates. The Rateable value represents the Agency's estimate of the open market annual rental value of a business/ non-domestic property; i.e. the rent the property would let for on the valuation date, if it were being offered on the open market. The rateable value is estimated based on the varying rents at the vicinity of a property. This represents a reasonable level of open market rental value, taking into account the expected turnover of the premise, the size, age and condition of the property, the length of the frontage, the depth and vertical layout, the visibility, the footfall and pedestrian flow volumes on surrounding streets etc. Because the rateable value of a property is used to determine the non-domestic property tax (business rate), and the evaluators have access to detailed information (such as contracts, revenue documents etc.), and because of this comprehensive evaluation documentation and methodology, rateable value is considered a very good indicator of the property value of a hereditament. The agency publishes a detailed set of information for each property; this includes the classification of its main use (detailed breakdown into more than 100 classes), the full address and postcode, the total area of the premise, the total rateable value and breakdown into zones with different rateable value per square metre, and the weighted average rateable value per square metre. This makes it possible to create a detailed map of rateable value for any use. . As said

Category Description	Scat Code
SHOPS	249 , 155 , 152 , 210 , 154
HAIRDRESSING SALONS	1910 , 23
SHOWROOMS	251 , 249
KIOKS	243 , 249
CAR SHOWROOMS	42
RETAIL WAREHOUSES	235
SUPERSTORES	139 , 152
MARKETS	165

Table 1: In this table we show the categories we have selected in the VOA datasets with their relative Scat Codes.

the dataset contains information on more than 3 10^5 non-domestic activities in the area of London. These are labeled with a *scat code* which depends of the category they belong to. For this analysis we have selected only the ones which belong to retailing scat codes, which are shown in table 1. The data is downloadable at (https://voaratinglists.blob.core.windows.net/html/rlidata.htm).

Population Census Data. The population data comes from the UK 2011 Census of Population which can be accessed at https://www.ons.gov.uk/census/2011census. The data records the usual resident population on census day, which was the 27th of March 2011. For the UK 2011 Census Workday population, the usually resident population is re-distributed to their usual places of work, while those not in work are recorded at their usual residence.

Social Medi9a Spatiotemporal Profiles: Foursquare. We have used one passively generated dataset. The dataset contains all venues listed on the website Foursquare.com within the M25 motorway which defines London's frontier. These amount to around 300 thousands different venues. Each record contains location coordinates, number of check-ins and unique visitors since venue was registered and detailed venue category (activity type). From all the entries in the Dataset we have filtered those belonging to the same categories in table 1. The Foursquare venue data was collected in December 2014.

2 Derivation of Logit Model

In this section we will go through Mc Faddens' solution of the Logit model [2], we have used in the formulation of

$$p_{i\to r} = \frac{A_r \exp\left(-\beta\, c(d_{ir})\right)}{\sum_{r'} A_{r'} \exp\left(-\beta\, c(d_{ir'})\right)} \tag{1}$$

The utility that a decision maker i obtains from choosing alternative r over other possibilities $r\prime$ is decomposed into a an observable known part v_{ir}, and an unknown part ω_{ir} we will treat as random. The utility therefore looks like

$$u_{ir} = v_{ir} + \omega_r \;\; \forall r \tag{2}$$

The probability that i chooses r over all other possibilities corresponds to the probability of that specific utility being greater than all others, namely

$$p_{i\to r} = \left(v_{ir\prime} + \omega_{r\prime} < v_{ir} + \omega_r \forall r\prime\right) = p\left(\omega_{r\prime} < \omega_r + v_{ir} - v_{ir\prime} \forall r\prime\right) \tag{3}$$

We have to now make some assumptions on the distribution of the random part ω in order to solve eq.(3). Following Mc Fadden's work we assume an identical independent extreme value, or Gumble distribution, which looks like

$$f(\omega) = \mathrm{e}^{-\omega}\, \mathrm{e}^{\mathrm{e}^{-\omega}} \tag{4}$$

and which in its cumulative form becomes

$$F(\omega) = \mathrm{e}^{\mathrm{e}^{-\omega}} \tag{5}$$

Exploiting the form in eq.(5) the solution to eq.(3) for a fixed $r\prime$ becomes

$$p_{i\to r} = \mathrm{e}^{\mathrm{e}^{-(\omega_r + v_{ir} - v_{ir\prime})}} \tag{6}$$

and given the independence assumption, we can extend the solution to all other alternatives $r\prime$ just by multiplying

$$p_{i\to r}|\omega_r = \prod_{r\prime \neq r} \mathrm{e}^{\mathrm{e}^{-(\omega_r + v_{ir} - v_{ir\prime})}} \tag{7}$$

which is the probability of i choosing r for a fixed value of ω_r. Now by integrating eq.(7) over the random element ω_r we obtain

$$p_{i\to r} = \int \left(\prod_{r\prime \neq r} \mathrm{e}^{\mathrm{e}^{-(\omega_r + v_{ir} - v_{ir\prime})}} \right) f(\omega_r) d\omega_r \tag{8}$$

which given the chosen form of $f(\omega)$, is in close form and is resolvable. After some algebra (see [1] section 3.10) we get to the form

$$p_{i\to r} = \frac{\mathrm{e}^{v_{ir}}}{1 + \mathrm{e}^{v_{ir}}} \tag{9}$$

which is the logit choice probability. Finally the utility is usually expressed as linear in its parameters (see [1] for an exhaustive analysis), namely $v_{ir} = \beta x_{ir}$, where x_{ir} is a vector of observed parameters relating to the alternative r, and β is the inverse of the standard deviation of the distributon. For a utility linear in its parameters, the logit probabilities becomes

$$p_{i\to r} = \frac{\mathrm{e}^{\beta x_{ir}}}{\sum_{r\prime} \mathrm{e}^{\beta x_{ir\prime}}} \tag{10}$$

Now wanting to apply this equation to the retail location choice, we start by defining the utility in the form

$$x_{ir} = u_p - p_r - c(d_{ir}) \tag{11}$$

which corresponds to the utility of acquiring the product p, the price at which the product is sold by retailer r and the generalised cost of travelling from i to r. In our model the consumer, of decision maker, faces the choice of which retailer to shop from. The probability of choosing retailer r therefore, by substituting eq.(11) in eq.(10) can be expressed as

$$p_{i\to r} = \frac{\exp\left(-\beta_i(u_p - p_r - c(d_{ir}))\right)}{\sum_{r'} \exp\left(-\beta_i(u_p - p_{r'} - c(d_{ir'}))\right)} \tag{12}$$

as found in eq.(2) in the manuscript.

References

[1] Train, K.*"Discrete Choice Methods with Simulation"* Cambridge University Press, (2009).

[2] McFadden, D. *"Conditional logit analysis of qualitative choice behavior"* Institute of Urban and Regional Development, University of California, (1973).
